# Impact of cold storage on the oxygenation and oxidation reactions of red blood cells

**DOI:** 10.3389/fphys.2024.1427094

**Published:** 2024-08-19

**Authors:** Tigist Kassa, Sirsendu Jana, Jin Hyen Baek, Abdu I. Alayash

**Affiliations:** Laboratory of Biochemistry and Vascular Physiology, Division of Blood Component and Devices, Center for Biologics Evaluation and Research, Food and Drug Administration, Silver Spring, MD, United States

**Keywords:** RBC storage lesion, oxygen binding, oxidation reactions, hemoglobin, band 3

## Abstract

**Introduction:** Electrostatic binding of deoxyhemoglobin (Hb) to cytoplasmic domain of band 3 anion transport protein occurs as part of the glycolytic regulation in red blood cells (RBCs). Hb oxidation intermediates not only impact RBC’s oxygenation but also RBC’s membrane through the interaction with band 3. It is not known however whether these critical pathways undergo changes during the storage of RBCs.

**Methods and Results:** Oxygen parameters of fresh blood showed a sigmoidal and cooperative oxygen dissociation curve (ODC) for the first week of storage. This was followed by a large drop in oxygen affinity (P_50_) (from 30 to 20 mmHg) which remained nearly unchanged with a slight elevation in Bohr coefficients and a significant drop in extracellular acidification rates (ECAR) at the 42-day storage. Oxidation of Hb increased with time as well as the formation of a highly reactive ferryl Hb under oxidative stress conditions. Ferryl Hb interacted avidly with RBC’s membrane’s band 3, but to lesser extent with old ghost RBCs.

**Discussion:** The observed alterations in RBC’s oxygen binding may have been affected by the alterations in band 3’s integrity which are largely driven by the internal iron oxidation of Hb. Restoring oxygen homeostasis in stored blood may require therapeutic interventions that target changes in Hb oxidation and membrane changes.

## Introduction

The primary aim of blood transfusion is to maintain and/or to restore oxygen supply to various organs. Earlier studies showed that blood stored for more than a few days, for example, in acid-citrate dextrose, exhibited a significant increase in oxygen affinity, resulting in a left shift in the oxygen dissociation curves (ODCs) (smaller P_50_) (P_50_ is the partial pressure of oxygen at which 50% of Hb is saturated with oxygen). This is in large part due to changes in the Hb molecule (Gullbring and Strom, 1956). Transfusion of abnormally high oxygen affinity Hb could have significant consequences in a patient whose capability for physiologic adaptations may be severely compromised (Bunn et al., 1969). Further advances in the storage of red cell donations were made possible with the introduction of phosphates and adenine, which allowed for a longer storage period of whole blood units. The additive solutions that extend the storage period also preserve the quality of the red blood cell concentrate during storage (Hess, 2006; D'Alessandro et al., 2010).

Stored RBCs undergo biochemical and morphological changes, which results in what is known as the storage lesion (Scott et al., 2016). Morphological changes have been reported to include echinocytosis, spherocytosis, and vesiculation (microparticle formation (MPs), and these are among the most frequently reported changes during the storage of blood (Antonelou et al., 2012). Lactic acid accumulation and a decrease in RBC pH; loss in 2,3-diphosphoglycerate (2,3-DPG), ATP, and intracellular potassium; and increases in calcium and oxidative damage are among the most impacted biochemical parameters (Hess, 2014). Consequently, RBCs experience a loss of 2,3-DPG and subsequent decreases in the P_50_ of Hb, which, in turn, decreases peripheral oxygen unloading (Doctor and Spinella, 2012).

Another important aspect of oxygen homeostasis within RBCs that can be impacted is the normal interactions of deoxyHb with the cytosolic domain of transmembrane band 3 protein, also known as anion exchanger 1 (AE 1), which together regulate the activity of glycolytic enzymes (Issaian et al., 2021). The signaling function of membrane-bound deoxyHb and the structure of the docking sites in the cytosolic domain of band 3 protein are well documented (Livshits et al., 2023). It was estimated that in RBCs from healthy donors, approximately 0.5%–12% of the Hb forms is membrane-bound, whereas the rest of the Hb remains in the cytosol (Kosmachevskaya and Topunov, 2018).

Several factors were found to influence the levels of Hb-band 3 complex formation, including oxyHb saturation, oxidative stress, cellular Hb concentration, and elevated Hb oxidation to methemoglobin (Puchulu-Campanella et al., 2013). This suggests that oxidation of heme iron is an important internal reaction that is linked to band 3 structural proteins. We have recently proposed a model describing the effects of Hb-dependent oxidation reactions on band 3 and other membrane proteins based on our work on normal and sickle RBCs. A redox transition of Hb into higher oxidation states (ferrylHb) through its pseudoperoxidative cycle interacts with band 3, resulting in oxidative modifications of the band 3 network of proteins, which can also promote post-translational changes, for example, phosphorylation (P) and ubiquitination (Ub) of Hb itself (Jana et al., 2018; Jana et al., 2023). As hemin may be liberated from oxidized Hb under pathological conditions, these hemin-induced effects on spectrin, protein 4.1, and membrane skeletal stability have been reported to play a role in the membrane lesion of these RBCs (Welbourn et al., 2017).

The phosphorylation of RBC’s cytoskeletal and membrane proteins is considered essential for microparticle (MP) formation, and band 3 is one of the most important membrane proteins that undergo extensive phosphorylation under many pathological conditions (i.e., sickle cell disease) and during RBC storage (Barshtein et al., 2021; Jana et al., 2023). These phosphorylation processes (mostly at tyrosines) act essentially as redox sensors for RBCs (Brunati et al., 2000).

We have also recently reported changes during storage in total band 3 levels, as measured by immunoblot analysis using an anti-phospho tyrosine antibody. A progressive increase in the phosphorylation status of band 3 with the increasing storage time in both RBCs and their derived MPs was observed. There are multiple phosphorylation sites on band 3, and one of the most notable is tyrosine 359 (Jana et al., 2023).

The question that remains, however, is to what extent the age of RBCs influences the degree of band 3 phosphorylation and whether these changes are somehow linked to alterations in RBC’s oxygen-carrying capabilities. In this report, we focused our efforts on the interplay between RBC’s oxygenation and oxidation reactions and the impact of storage on these pathways and on the integrity of band 3 proteins. We monitored these reactions before and after the storage of blood for 42 days under similar blood banking storage conditions. As expected, there was a considerable shift in oxygen affinity parameters toward Hb’s higher oxygen binding affinity with minor alterations in Hb cooperativity and responses of RBCs toward pH changes (Bohr effects) and ECAR. These changes were also reflected to lesser extent in the RBC’s deformability and elongation index, which is a reflection of RBC’s membrane flexibility during storage for 42 days.

## Materials and methods

### Materials

All chemicals and reagents were purchased from Sigma-Aldrich (Saint Louis, Missouri) or Thermo Fisher Scientific (Pittsburgh, Pennsylvania) unless otherwise specified. Blood samples used in this study were obtained from healthy donors from the National Institute of Health (NIH) Blood Center, Bethesda, Maryland (FDA/CBER, IRB protocol 03084B). Hydrogen peroxide (H_2_O_2_) (30% w/w) was purchased from Sigma-Aldrich. Fresh solutions of H_2_O_2_ were prepared for every experiment from a stock solution by making appropriate dilutions in deionized water, and the concentration of H_2_O_2_ was determined spectrophotometrically at 240 nm using a molar extinction coefficient of 43.6 M^-1^cm^-1^. Buffer solutions were prepared by mixing monobasic and dibasic potassium phosphate dissolved in deionized water, and the pH was adjusted appropriately.

### Spectrophotometry

Spectrophotometric measurements were carried out in a UV-visible diode array spectrophotometer (Agilent HP 8453). The levels of ferrous, ferric, and ferryl Hbs were measured based on the absorbencies at λ = 541, 576, 630, and 700 nm using published extinction coefficients (Meng and Alayash, 2017). Hb concentrations were calculated based on heme concentration.

Fresh whole blood (50 mL) was first leuko-reduced, and RBCs were then separated from plasma by centrifugation. After removing the plasma and buffy coat, packed RBCs were stored at 4°C in a red-cell storage bag containing 11 mL of AS-3 storage solution for 42 days. A measure of 1 mL of the stored blood was taken at specific times (0 day and 42 days) for photometric analyses. The solution was then washed with 2 mL phosphate-buffered saline (PBS), gently stirred, and then centrifuged for 5 min at room temperature. The supernatant was removed; this was repeated twice. RBCs were lysed by adding 3 mL of water, gently stirred, and left to stand for 10 min at room temperature. A total of 24 mg NaCl was added to the lysate, and the lysate was centrifuged at 4,000 × g for 10 min. The supernatant was filtered with a 0.2-μM filter to remove the RBC membrane. The solution was concentrated, and Hb concentration was measured.

### Autoxidation measurements

Autoxidation experiments were carried out by incubating Hb/RBC samples in 50 mM phosphate buffer at pH 7.4 at 37 °C for 24 h. Absorbance changes in the range of 350–700 nm due to the spontaneous oxidation of Hb (60 μM in heme) were recorded in a temperature-controlled photodiode array spectrophotometer (HP-8453). Multicomponent analysis was used to calculate the oxyHb and metHb based on the extinction coefficients of each species (Meng and Alayash, 2017).

### Hydrogen peroxide-mediated oxidation and ferryl hemoglobin formation

Spectral changes due to the H_2_O_2_-mediated reaction of RBCs/Hb (60 μM, heme) were monitored by a photodiode array spectrophotometer using H_2_O_2_ (20 mM) in a total of 1 mL solutions at room temperature for 5-min incubation times. Ferryl Hb formation was followed by monitoring characteristic absorbance changes over time in the visible region (Kassa et al., 2017). For the verification of the ferryl intermediate, 2 mM sodium sulfide (Na_2_S) was added to transform ferryl Hb to sulfhemoglobin (sulfHb). The formation of the sulfHb can be monitored by the appearance of an absorbance band at 620 nm and was estimated using the published extinction coefficient (Berzofsky et al., 1971).

### Oxygen equilibrium studies

Oxygen dissociation curves (ODCs) for RBCs were obtained using the Hemox Analyzer (TCS Scientific, New Hope, PA). The ODCs of suspensions of cells were determined as previously described (Gelderman et al., 2010). To measure ODCs, samples from the stored blood were taken, washed, packed, and resuspended in plasma to a hematocrit of approximately 25%. A suspension of ∼120 μL was added to 3 mL of Hemox buffer, at pH 7.4, in a cuvette and subjected to ODC analysis at 37°C. A computer-based analysis of oxygen-binding curves was performed, yielding P50 (partial pressure of oxygen at which Hb is half saturated) and n_50_ (Hill coefficient) for oxygen binding.

Dependence of the oxygen affinities of the Hbs on pH (Bohr effect) was carried out as in the previously published procedure (Jia and Alayash, 2009). Three different pH buffer solutions were prepared (6.8, 7.4, and 7.6) at room temperature in 0.5 M HEPES.

#### RBC ghost-membrane preparation

RBC ghosts were prepared by repeated washing of RBCs using a hypotonic solution followed by a ghost-membrane preparation method published earlier (Himbert et al., 2017).

### Measurements of ATP, 2,3-DPG, and protein carbonylation

Intracellular ATP level within RBCs (fresh and stored) was measured using a luminometric ATP-assay kit (ab113849) obtained from Abcam (Abcam, Cambridge, MA, USA) following methods previously published (Cloos et al., 2020; Jana et al., 2023). Luminescence was measured using a BioTek Synergy HTX microplate reader (Agilent, Santa Clara, CA, United States) with a proper blank control to minimize Hb interference.

Intracellular 2,3-DPG levels were measured using a commercial Roche diagnostic kit (10148334001) purchased from Sigma Aldrich (Sigma-Aldrich, Saint Louis, MO, United States) following a method published before by Płoszczyca et al. (2021). The 2,3-DPG assay is based on enzymatic cleavage of 2,3-DPG and oxidation of nicotinamide adenine dinucleotide. All the 2,3-DPG levels were normalized to the corresponding hematocrit values.

Protein carbonyl content was assessed as a measure of intracellular protein oxidation in RBC lysates by an assay kit (ab126287, Abcam, Cambridge, MA, United States) using dinitrophenyl hydrazine (DNPH), where carbonyl groups in protein side chains are derivatized to DNP-hydrazone following a reaction with DNPH (Jana et al., 2023). The absorbance of DNP hydrazones formed in this reaction was measured at 375 nm using a BioTek Synergy HTX microplate reader (Agilent, Santa Clara, CA, United States).

#### Hemoglobin binding and co-immunoprecipitation assays of band 3

We have previously shown that Hb oxidation promotes complex formation with band 3 (Jana et al., 2018). To assess the effect of Hb oxidation on complex formation with band 3 protein in fresh and aged RBCs, we utilized Hb-free RBC ghost membranes (0 day and 42 days) and then incubated the membrane (20 µg) with either Hb^2+^ or Hb^4+^ (50 µM) for 2 h at ambient temperature (25°C). Following incubation, the reaction mixture was washed with hypotonic PBS (1: 10 diluted with water) three times by centrifugation at 15,000 × g, discarding the Hb-rich supernatant in each wash. Band 3-Hb complex formation in RBC ghost’s membrane was analyzed by co-immunoprecipitation assay using a commercial protein G immunoprecipitation kit purchased from Sigma-Aldrich (Sigma-Aldrich, Saint Louis, MO, United States), as described earlier (Jana et al., 2018). For co-immunoprecipitation of human band 3 protein, a polyclonal rabbit anti-band 3 antibody was purchased from United States Biological (band 3 Antibody (A-6): sc-133190 Catalog No. 303147–100 ug). Briefly, the samples were pre-cleared using protein G agarose beads for 2 h at 4°C and then incubated overnight at 4°C with bait antibody (band 3) in 0.5X IP buffer supplied in the protein G immunoprecipitation kit. The beads were then washed four times with 1X IP buffer and finally two times with PBS. Proteins were eluted from the protein G agarose beads using 100 µL NuPage LDS sample buffer containing the reducing agent (Thermo Fisher Scientific). Eluted immunoprecipitates were resolved by SDS–PAGE using 4%–12% bis-tris gels and analyzed for Hb protein binding by Western blotting, as described earlier (Jana et al., 2018).

### Gel electrophoresis and immunoblotting

RBC cell lysate proteins were resolved by SDS–PAGE using precast 4%–20% NuPAGE bis-tris gels (Thermo Fisher Scientific, Waltham, MA) and then transferred to PVDF membranes using an Invitrogen iBlot 2 Gel Transfer Device gels (Thermo Fisher Scientific, Waltham, MA). PVDF membranes were processed with different specific primary antibodies, for example, anti-band 3 (ab108414), anti-phospho (Y359) band 3 (ab77236), anti-hemoglobin (ab19363), and anti-β actin (ab8227) (Abcam, Cambridge, MA, United States). Appropriate HRP-conjugated goat anti-mouse IgG (ab97040) and anti-rabbit IgG (ab205718) secondary antibodies were also obtained from Abcam (Cambridge, MA).

### Glycolytic rate measurement in RBCs

Glycolytic rate in RBCs was assessed in real time using an Agilent Seahorse XF24 Extracellular Flux analyzer (Agilent, Santa Clara, CA) following a protocol published by Wu et al. (2023) with some modifications. Briefly, RBCs from storage bags were washed twice with glucose-free XF-base assay medium (Agilent, Santa Clara, CA) by centrifugation at 2,500 × g at ambient temperature. Then, 5 × 10^6^ RBCs per well were plated onto XF24 cell plates coated with Cell-Tak (354242, Corning, New York, United States) suspended in glucose-free 100 μL XF-base assay medium. After seeding, the cell plates were centrifuged for 5 min at 1,000 × g at room temperature. An amount of 400 μL of XF base-assay medium was gently added to each well without disturbing the attached RBCs. Extracellular acidification rate (ECAR) was measured by serial injections of glucose (10 mM) and glycolytic inhibitor 2-deoxy-glucose (2-DG, 100 mM), as described previously and following the manufacturer’s protocol (Jana et al., 2023). For each group, no glucose or other additives were added in three identical wells with RBCs for background corrections to eliminate any interference by Hb. Two major glycolytic parameters, for example, maximum glycolysis and glycolytic reserve capacity, were calculated from the ECAR plots. Maximum glycolysis was obtained from the difference between basal ECAR before glucose addition and maximum ECAR achieved after the glucose addition. The glycolytic capacity was the difference between maximum ECAR achieved by glucose and the minimum ECAR after the addition of 2-DG.

### RBC deformability test

RBC deformability was determined at various fluid shear stresses by laser diffraction analysis using an ektacytometer (LORRCA MaxSis, RR Mechatronics, Hoorn, The Netherlands). Shear stress–elongation index (EI) curves were obtained at 37°C at nine shear stresses between 0.3 and 30 Pa according to manufacturer’s instructions. Briefly, 25 μL of pre-oxygenated blood was added to 5 mL of an Elon-ISO solution with a mean viscosity of 27.64 mPa s (Mechatronics). After the application of 1 mL of the RBC suspension, a laser beam was directed through the sheared sample, and the diffraction pattern produced by the deformed cells was analyzed. Based upon the geometry of the elliptical diffraction pattern, the elongation index (EI) was calculated as follows: EI = (L − W)/(L + W), where L and W are the length and width of the diffraction pattern, respectively. An increased EI at a given shear stress indicated greater cell deformation and hence greater RBC deformability.

## Results

**FIGURE 1:**
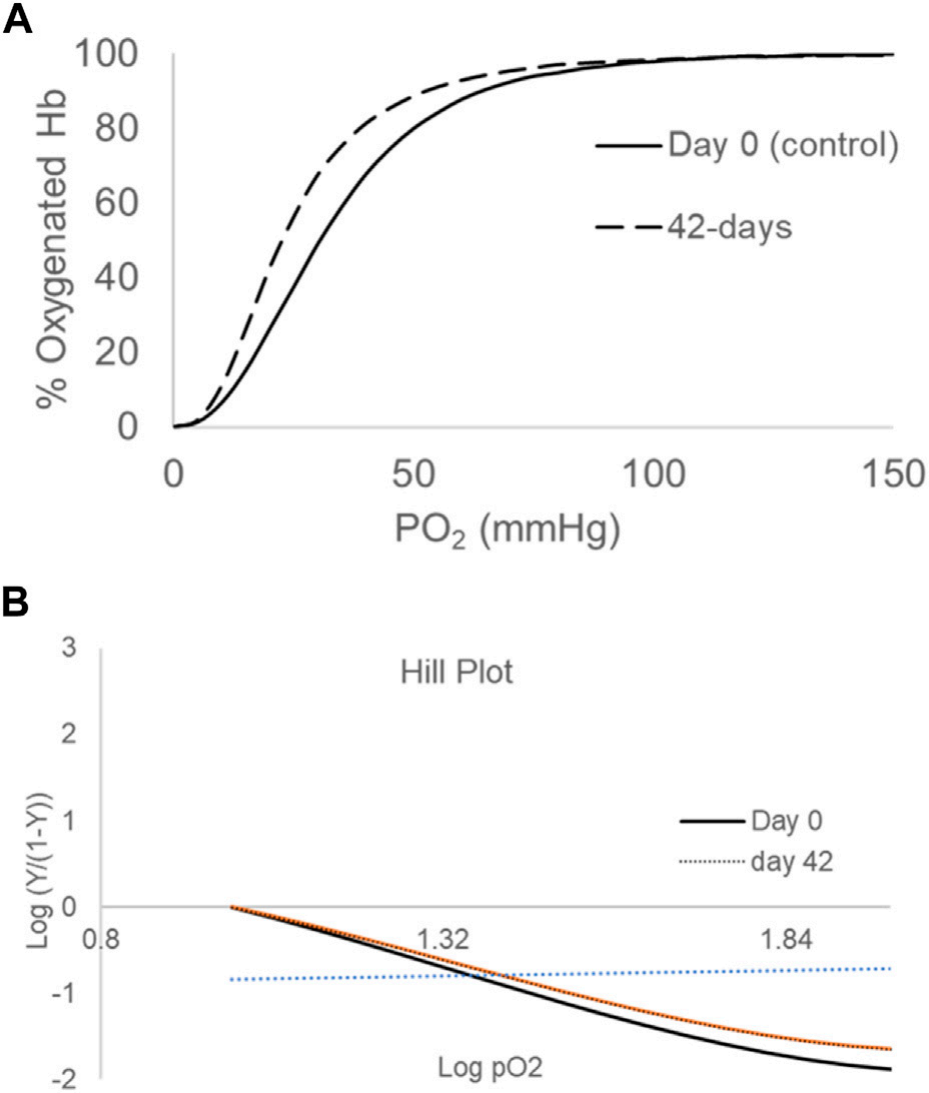
Oxygen-dissociation curves (ODCs) for blood stored over a period of 42 days **(A)**. ODCs were measured using the Hemox Analyzer (TCS Scientific). Experiments were carried out with 25% of hematocrit in 3 mL of Hemox solution (135 mm NaCl, 30 mm TES [N-Tris (hydroxymethyl)methyl-2-aminoethanesulfonic acid], 5 mm KCl} (TCS Scientific)) and anti-foaming agent at 37°C. The Hill plot (logy/1-y) vs. log (PO2) is shown in **(B)**. The plots are representatives of three separate measurements from the same blood bag (N = 3).

Figure 1A illustrates typical oxygen dissociation curves (ODCs) for blood at day-0 and day-42 in AS-3 solutions at 37°C under similar experimental conditions (pH 7.4, 5% CO_2_). At 42 days, there was no change in the shape of the curve, with it still being sigmoidal, but there was a clear shift to the left in the entire curve. The ODC also maintained a complete degree of saturation at high PO_2_ (100–150 mmHg). The P_50_ estimated from these curves are reported in Table 1, together with a slightly reduced Hill number (*n*), which reflects the degree of cooperativity among the four heme centers. The Hill plot (logy/1-year) vs. logPO_2_) is shown in Figure 1B. The drop in P_50_ from 30 to 20 mmHg with time is similar to the early reported values, including the most recent serial oxygen equilibrium studies (Gelderman et al., 2010; Jana et al., 2023). It is still not clear how such a drop in oxygen-carrying capabilities of RBCs translates in an *in vivo* setting. However, we have previously shown that a correlation exists between lowered P_50_s and hypoxia inducible factor (HIF-1α), an oxygen sensor measured in cell culture media containing aged RBCs (Jana et al., 2023) and in animals (Manalo et al., 2008).

**TABLE 1:** Oxygenation, oxidation reactions, and hemolysis in stored RBCs.

Sample	Day-0 (control)	Day-42
Hemolysis %	0.096 ± 0.02	1.68 ± 0.12
OxyHb %	100	99 ± 1.0
metHb% (25°C)	0	1 ± 1.0
metHb% (37°C)	31.1 ± 0.32	35.7 ± 1.62
p50 (mmHg)	30 ± 0.09	22.1 ± 0.21
n_50_ (Hill coefficient)	2.72 ± 0.05	2.54 ± 0.18
ATP (nmoles/mg Hb)	3.17 ± 0.31	2.31 ± 0.42*
2,3 DPG (nmoles/mg Hb)	11.8 ± 2.4	2.9 ± 1.9*
Bohr Coefficient (Tetramer) (H^+^)	−0.5	−0.7

Hemolysis was monitored spectrophotometrically by quantifying free hemoglobin in solutions (Jana et al., 2023). Autoxidation of hemoglobin RBCs (60 µM) in AS-3 solutions at pH (7.4) and room temperature followed for 42 days. Spectral analysis in the visible region of RBC solutions was followed spectrophotometrically in an Agilent spectrophotometer. Oxygen dissociation curves (ODCs) and the Hill coefficient of RBCs were obtained using the Hemox Analyzer™ (TCS, scientific, New Hope, PA). Samples from the stored blood were taken, washed, packed, and re-suspended in plasma to a hematocrit of approximately 25%. Approximately 120 μL of each suspension was added to 3 mL of Hemox buffer, pH 7.4, in a cuvette and subjected to ODC analysis at 37°C. The Bohr coefficients were derived from the slopes (ΔlogP50/ΔpH) for fresh and old RBCs (per Hb tetramer).

*p<0.01 vs. respective 0-day controls (N = 3).

Next, we assessed the responses of Hb’s oxygen affinity to changes in blood pH (Bohr effect) as a function of RBC’s age (Duhm, 1976; Jensen, 2004). Although it is generally assumed that the shift in ODC by changing blood pH has little or no effects on oxygen transport, more recent work showed that the Bohr effect of blood can change markedly in diabetes mellitus (Ditzel, 1976), chronic hypercapnia (Messier and Schaefer, 1971), and hypoxia.

**FIGURE 2:**
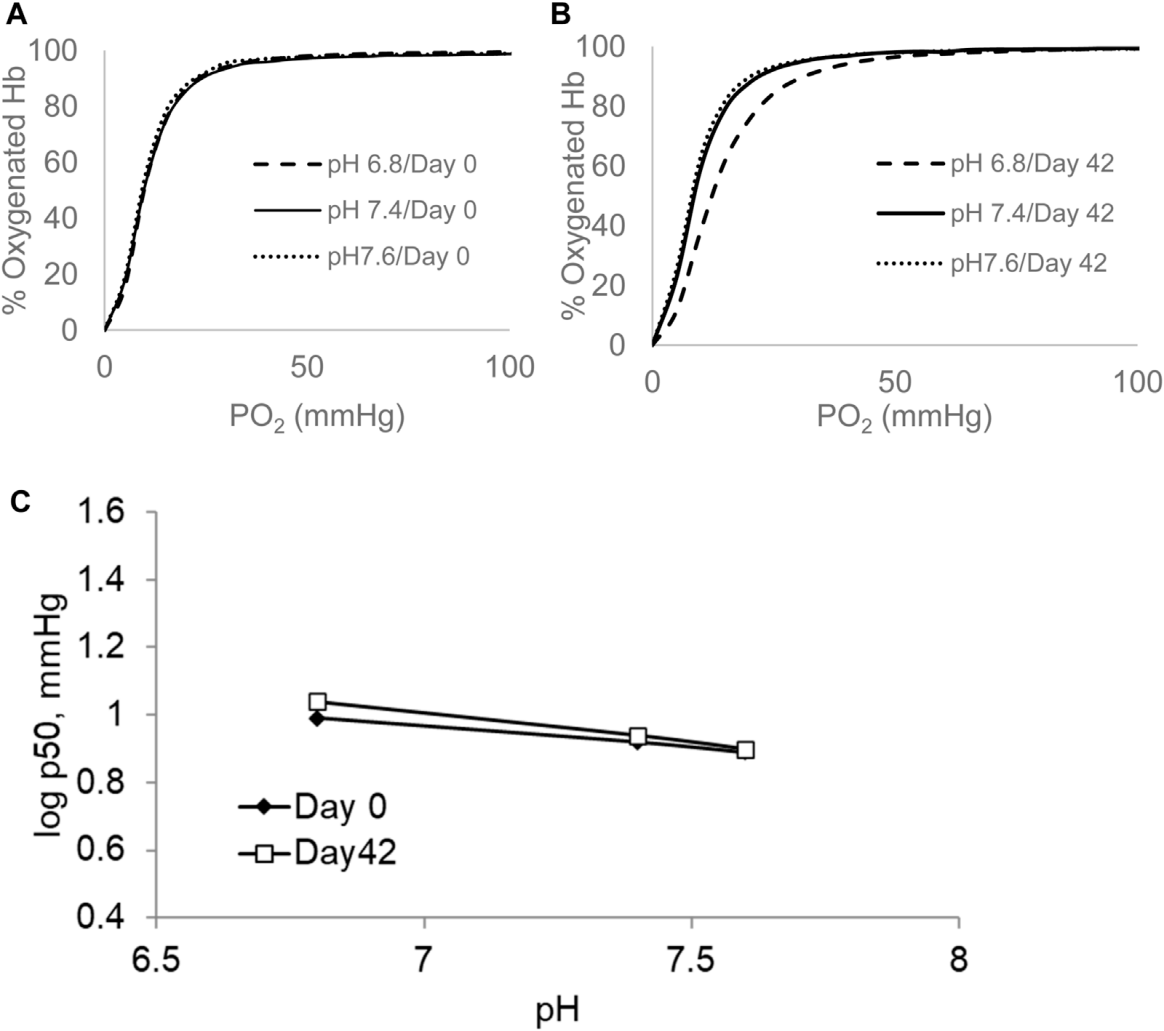
ODCs measured at different pHs for blood stored over a period of 42 days. The ODCs were measured in the Hemox Analyzer. P_50_ was measured at pH 6.8, 7.4, and 7.6 in 0.5 M HEPES at temperature of 37°C (0-day sample **(A)** and 42-day sample **(B)**). Bohr coefficient was derived from the slopes of log P_50_ *versus* pH **(C)**. All plots shown in this figure are representatives from three separate experiments (N = 3).

Figure 2A shows ODCs of RBCs under similar experimental settings as before (0.5 M HEPES buffer, using air that consists of 21% oxygen, 79% nitrogen, and 0.001% CO_2_ at 37°C) under three different pHs: slightly acidic, pH 6.8; normal physiological blood, pH of 7.4; and slightly basic, pH 7.6, respectively. The Bohr effect, which is defined as ΔlogP_50_/ΔpH, was determined in fresh (0-day) and old (42-day) blood. Unlike the case with control samples (Figure 2A), there was a clear right shift in the curve at the acidic pH in older RBCs (Figure 2B). The linear parts of the sigmoidal Bohr curves for RBCs from both groups are shown as a comparison at these three pHs in Figure 2C. The slopes derived from these lines were 2.0 and 2.8 for days 0 and 42, respectively, translating into Bohr coefficients of −0.5 and −0.7 (protons) (H^+^) that are exchanged for one oxygen molecule per tetramer in 0- and 42-day RBCs, respectively. These findings are consistent with those of early reported studies on fresh and stored blood (Högman, 1971). Bohr coefficients measured for RBCs and unlike free Hb are influenced by other allosteric effectors such as 2,3 DPG and ATP (Duhm, 1976). We have analyzed the basal ATP and 2,3 DPG levels in stored RBCs (42 days) (Table 1). The levels of both metabolites fell significantly during the storage period, with 2,3 DPG showing a more drastic fall (∼85%) than ATP (∼70%).

**FIGURE 3:**
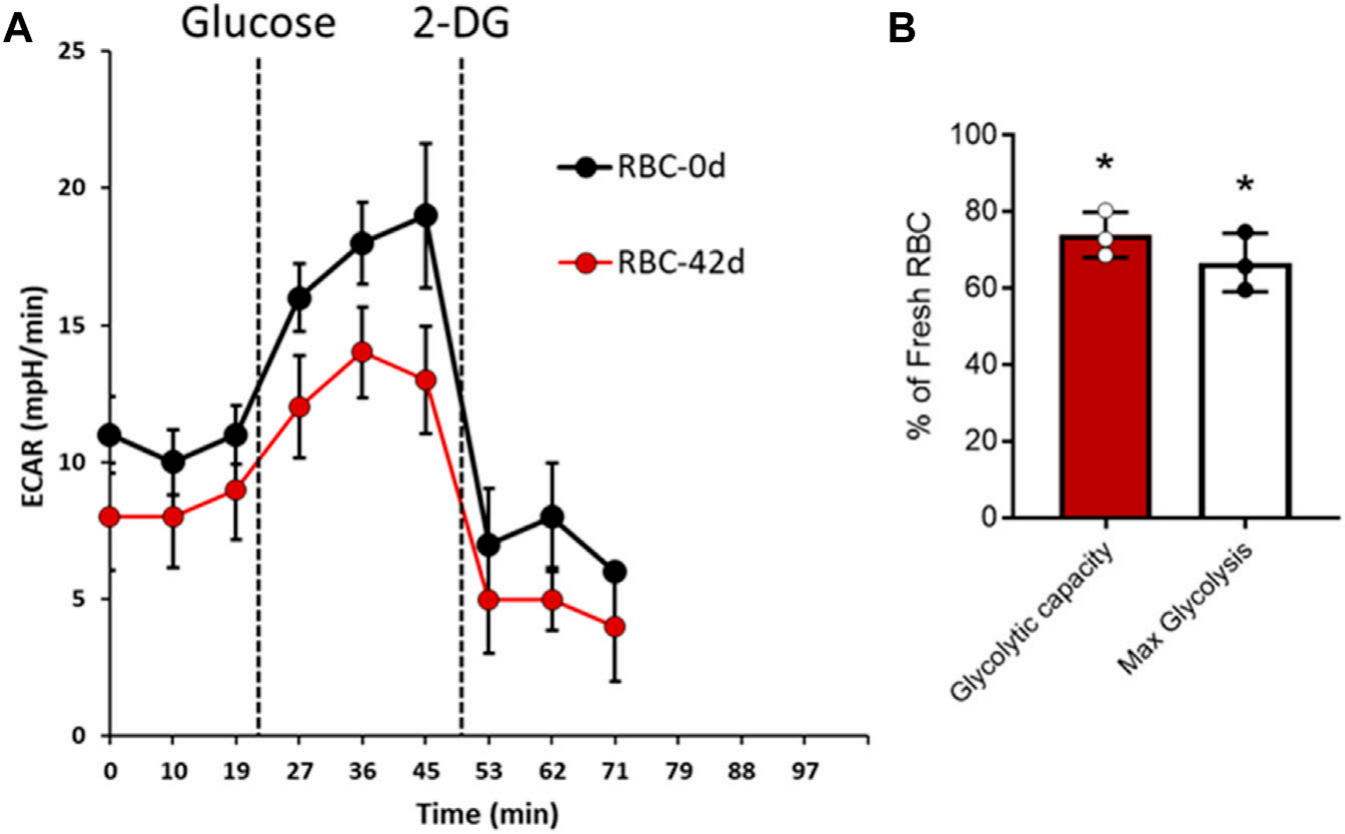
Glycolysis in fresh and stored RBCs. Extracellular acidification rate (ECAR) was measured in real time using XF24 extracellular flux analyzer for fresh (0-day) and stored (42-day) RBCs. **(A)** Representative plots showing average ECAR (mpH/min) values obtained from three identical replicate wells using one set of 0-day and 42-day RBC samples following addition of glucose and glycolytic inhibitor 2-DG. The ECAR experiments were repeated with three separate set of samples (N = 3). **(B)** Average glycolytic reserve capacity and maximum glycolysis were calculated from three separate sets of ECAR plots in 42-d RBCs, and the values were expressed as (%) of fresh RBCs. Statistical significance between means was calculated by paired Student’s t-test. *P < 0.05 vs. fresh RBCs are used as control.

To assess the impact of long-term storage on RBC’s metabolism, we carried out a novel experiment in which we were able to measure the extracellular acidification rate (ECAR) for RBCs that were seeded in a plate reader in real time using an Agilent Seahorse XF24 Extracellular Flux Analyzer (Agilent, Santa Clara, CA). This assay measures the proton flux from lactate generated by cellular glycolysis. Figure 3A shows that the addition of glucose in the media rapidly promoted glycolysis, as evident by the change in pH. However, the acidification rate was much lower in 42-day stored RBCs than that in fresh RBCs (Figure 3A). Maximum glycolysis and glycolytic capacity were calculated from the ECAR plot after the addition of glycolytic inhibitor 2-DG, which completely stopped the glycolysis within RBCs (Figure 3B). Figure 3B shows a significant difference in both reserve glycolytic capacity and maximum glycolysis in these two different age-groups, indicating a slower rate of glycolysis with older RBCs (Figure 3B).

**FIGURE 4:**
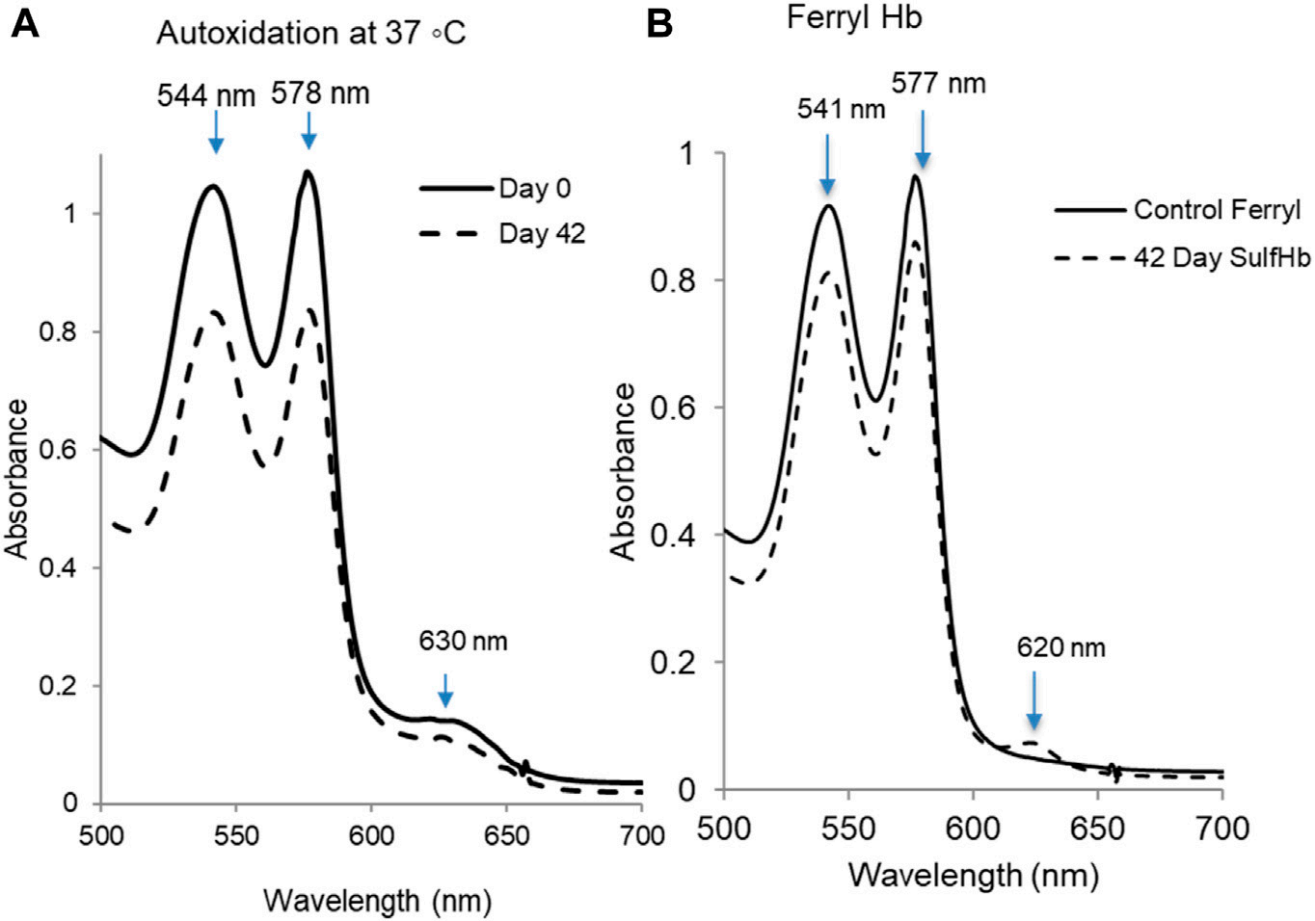
Autoxidation and peroxide-mediated oxidation of hemoglobin in stored RBCs. **(A)** Samples from stored blood at 0 and 42 days were collected and immediately hemolyzed. An amount of 60µM of hemoglobin samples were prepared, and the autoxidation was measured spectrophotometrically at 37°C. **(B)** Hydrogen peroxide-induced oxidation of 42-day sample of RBCs. The sample was hemolyzed, and 60 µM of hemoglobin was treated with 20 mM hydrogen peroxide, followed by the addition of 2 mM Na_2_S to derivatize the ferryl formed in the reaction. The solid line is ferryl Hb before addition of the Na_2_S. SulfHb spectrum obtained immediately after the addition of Na_2_S to oxidized HbA solution that exhibits a characteristic strong peak at 620 nm (dashed line). Plots shown in this figure are representatives of three separate experiments (N = 3).

During storage, the spontaneous lysis of a small fraction of red cells takes place, and vesicles containing both lipids and Hb from intact red cells are shed into the supernatant plasma (Chaudhary and Katharia, 2012). Under air-saturated conditions, the ferrous form of Hb (HbF^2+^) spontaneously oxidizes to a ferric (metHb) form (HbFe^3+^). Figure 4A shows typical kinetic spectral scans of the changes captured during incubation of 60 μm oxyHb in 50 mM potassium phosphate buffer (pH 7.4 at 37°C) for the samples throughout the 42-day study. Under room temperature, these autoxidation experiments showed little or no change in the spectra collected over the 42-day period. However, there was a steady decline in peaks in the visible region (544 and 576 nm) and a corresponding increase in the 630-nm peak, indicative of metHb formation at 37°C. Table 1 lists the amount of met formation in RBCs at 25°C and at 37°C. At the higher temperature, there was a considerable buildup of metHb in cells that were incubated at day 1 and more so at the end of the incubation period. Some degree of hemolysis was also noted at these two different conditions.

Peroxide reactions with both fresh and old RBCs were carried out at 37°C using 20 mM peroxide. There was slight change in spectral scan of oxyHb for the day 1 samples, but there was a considerable change in the spectra collected over the 42-day incubation (Figure 4B). During a typical experiment for the 42-day-old sample, the initial oxidation of ferrous heme was seen by a gradual loss of the α and β visible absorbance bands at 576 nm and 541 nm and the appearance of a ferryl intermediate with absorbance peaks at 545 and 580 nm, and a flatter region between 600 and 700 nm was seen. The ferryl peak then reverts over 30–60 min to metHb, with peaks at 541 nm, 576 nm, and 630 nm, completing a pseudoperoxidase cycle. Due to the transient nature of ferrylHb, we added Na_2_S to conjugate the ferryl heme into sulfHb, which absorbs at 620 nm (Figure 4B).

**FIGURE 5:**
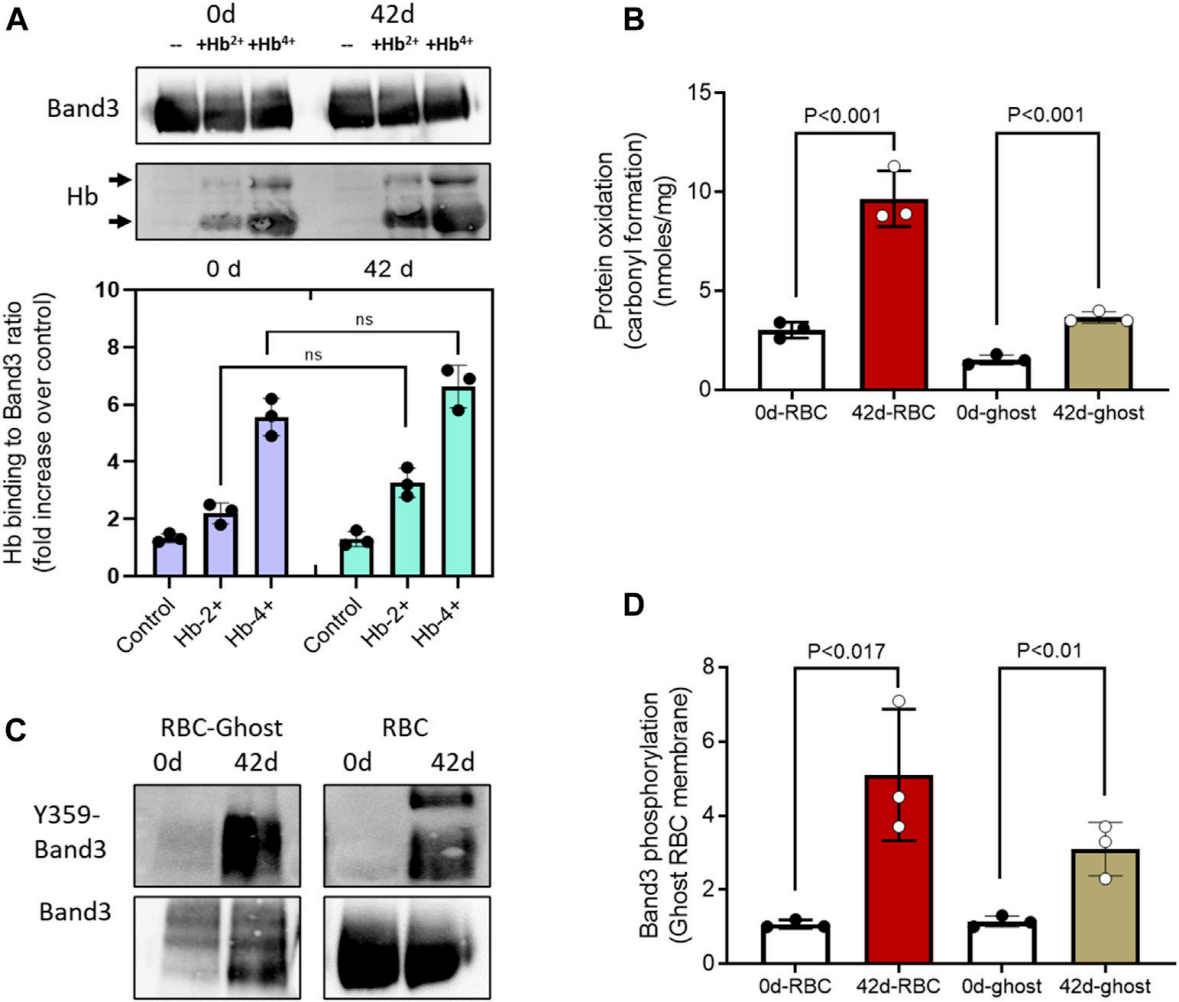
Post-translational changes in stored RBC membrane. **(A)** Co-immunoprecipitation of band 3 was carried out and probed with anti-Hb antibody. Band 3-Hb complex formation in RBC ghost’s membrane was analyzed following *in vitro* incubation with different Hb oxidation states using a co-immunoprecipitation assay and immunoblotted using anti-band 3 and anti-Hb antibodies (lower panel). Upper panel 90 KD bands show equal band 3 loading, and lower panel shows Hb binding developed with anti-Hb antibody. Immunoblots shown are representatives from three separate identical experiments (N = 3). Histograms showing the effect of oxidized (Hb4+) and unoxidized (Hb2+) hemoglobin binding to fresh (0-day) and stored (42-d) RBC ghost membrane-bound band 3. **(B)** Levels of protein carbonylation in RBC ghost membranes and RBC lysates from 0-d and 42-d samples (N = 3). **(C)** Immunoblots showing the levels of band 3 phosphorylation (Y359) in RBC ghost membranes and RBC lysates from 0-day to 42-d samples. Immunoblots shown are representatives from three separate identical experiments (N = 3). **(D)** Histogram showing phospho Y359-band 3/total band 3 ratio, fold change over fresh (0-d) RBC lysates, or RBC-ghost membranes.

To further assess the metabolic and biochemical changes in aged RBCs and their membranes under oxidative stress conditions (in the presence of ferryl Hb), we prepared RBC ghost membranes from fresh and aged RBCs and then analyzed for some of the post-translational modifications, for example, carbonyl oxidation of proteins and phosphorylation of band 3. Oxidized Hbs, especially the ferryl form, are known to bind with membrane-bound anion exchanger protein band 3 (Welbourn et al., 2017; Jana et al., 2018). To assess the susceptibility of band 3 protein binding with Hb, we incubated ghost membranes prepared from fresh and aged RBCs with non-oxidized (HbFe^2+^) and oxidized (HbFe^4+^) Hb for 1 h and then analyzed them by co-immunoprecipitation assay using the anti-band 3 antibody to capture any band 3-Hb complex. Figure 5A shows a marginal but non-significant increase in HbFe^2+^ binding with band 3 in either fresh or aged RBC-ghost membranes. In contrast, upon incubation with the highly oxidized ferryl form, a strong binding with band 3 occurred in both fresh and aged ghost preparations (Figure 5A).

Next, we analyzed the level of protein oxidation by measuring carbonylation in RBC and in ghost membranes from fresh and stored samples. We found a significant increase in protein oxidation in both preparations. However, due to the presence of Hb and other cytosolic proteins in intact RBCs, a higher level of carbonylated proteins was seen in RBC lysates than in ghost-membranes (Figure 5B). By using a specific anti-phospho (Y359) band 3 antibody, we also observed a high degree of phosphorylation of band 3 protein in both aged RBC lysates and ghost-membranes compared to their respective 0-day samples (Figures 5C, D).

**FIGURE 6:**
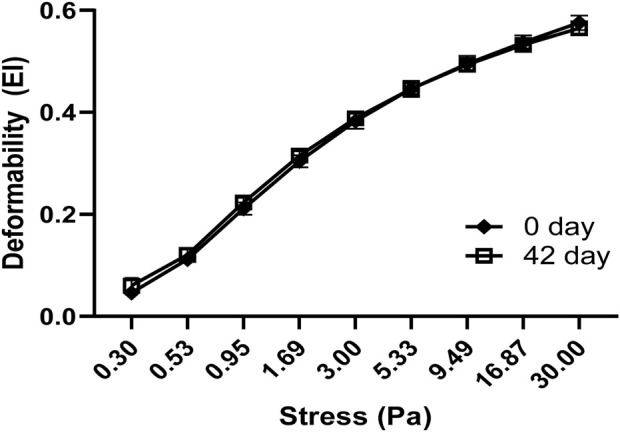
Comparison of red blood cell deformation curves in fresh and stored blood samples. RBC deformability was determined by plotting shear stress–elongation index (EI) curves obtained at 37°C at nine shear stresses between 0.3 and 30 Pa. Data are presented as mean ± SD, N = 4 for each group. There was no significant difference between the groups in two-way ANOVA analysis.

Oxidative stress and shear stress alter the ability of RBCs to deform and undergo changes in cell morphology, as well as RBC’s orientation. Normal human blood samples stored for 42 days at 4°C were used in the deformability studies. RBC deformability tests using fresh and stored blood samples were performed using the LORRCA MaxSis system. The deformability profile of stored blood samples showed little changes compared to fresh blood (Figure 6), but there was no significant difference between fresh and stored blood.

## Discussion

Despite recent advances in blood banking sciences, the therapeutic significance of alterations in the oxygen affinity of stored blood is not yet fully understood. It has been reported that it is unlikely that any untoward effect would be observed unless multiple transfusions were given to patients. In such cases, failure of Hb to unload sufficient oxygen to tissues due to its increased affinity could have important consequences (Bunn et al., 1969). Equally unclear is the impact of long-term oxidation of cytosolic Hb and its interaction with structural membrane proteins on the metabolic activity of RBCs as they circulate in blood. In this investigation, we focused our efforts on documenting the changes in oxygen binding and in the natural allosteric effectors of Hb and how these alterations impact the redox state of RBCs including the membrane proteins during the aging process of RBCs.

The oxygen dissociation data presented in this report were generated at normal physiological conditions (i.e., pH 7.4% and 5% CO_2_ at 37°C) using the automated Hemox analyzer. The Hemox analyzer is ideally suited to provide optimal measurements of whole-blood oxygen-carrying parameters with accuracy higher than other commercially available methods (Guarnone et al., 1995). This is in large partly due to the built-in dual-wavelength spectrophotometry, which minimizes optical interferences by RBCs (Guarnone et al., 1995). The data confirm earlier studies that showed that oxygen affinity increases during storage, resulting in a “shift to the left” of the ODCs. Although ODCs from day-0 and day-42 maintained their sigmoidal shape and reached saturation at high PO_2_, the decrease in P_50_ from 30 to 20 mmHg, however, is considerable. There are several possible factors that may have contributed to the “shift to the left” of the ODCs, including metHb, 2,3-DPG, ATP, and GSH (Bunn et al., 1969).

Our calculated p50 values from RBCs in HEMOX buffer (pH 7.4) were higher than those of similar reported experiments in BIS-Tris buffer (Rogers et al., 2024). The cause for the different effects of buffers on oxygen affinity of Hb/RBCs is well documented and may lie within small differences in NaCl concentrations contained in isotonic buffers such as HEPES, giving larger p50 values at lower NaCl concentrations (100 mM NaCl in BIS-Tris vs. 140 mM NaCl in HEPES) (Mawjood and Imai, 1999). Additionally, another reported advantage of using HEPES buffer for Bohr effects experiments is that HEPES allows for a more accurate assessment of Hb–oxygen affinity (Weber, 1992).

MetHb levels during the storage for 42 days and prior to ODC construction remained below 2%. It was estimated that approximately 20%–30% “inactive” Hb would be required to bring about the degree of increased oxygen affinity observed in stored blood (Bunn et al., 1969). Although the role of GSH in Hb’s oxygen affinity is not that clear, both DPG and ATP levels showed strong correlation with oxygen affinities in aged RBCs. It has been well established that ATP and 2,3 DPG levels within RBCs fall significantly under the storage conditions, and our results match with the previous findings (Karger et al., 2012).

During the 120-day, normal life span, approximately 1%–3% of Hb is transformed into an oxidized non-functional form (ferric/met). In relatively healthy and young RBCs, metHb is rapidly converted back to ferrous/oxyHb by metHb reductase in the presence of NADH. During the storage of RBCs, the enzymatic activity of metHb reductase is increasingly diminished, resulting in increased levels of metHb that is not converted back to oxyHb (Alayash, 2022). In addition, antioxidant machinery of RBCs is also diminished, resulting in the oxidation and deterioration of membrane lipids and proteins, which can ultimately lead to irreversible damage to the membrane. The temperature dependency of metHb reductase in humans and animal RBCs has been previously noted (Hlastala et al., 1977; Jensen and Nielsen, 2018), which may explain higher metHb formation at higher temperature, as seen in Table 1.

In this study, to correlate the low level of ATP seen in the 42-day-old RBCs, we have used the real time glycolytic rate monitoring as an indicator of impaired metabolism in stored RBCs. The use of extracellular flux analyzer to measure glycolysis in RBCs has been very limited, except a recent report where ECAR was used to successfully differentiate glycolytic capacity among high-altitude polycythemia population (HAPC) and low-land control (LLC) population in Tibet (Wu et al., 2023). This study emphasized the role of tyrosine phosphorylation of band 3 protein as a major influencing factor affecting glycolysis in RBCs. Band 3 has diverse biological functions being the most abundant protein in the RBC membrane and harbors binding sites for glycolytic enzymes (e.g., glyceraldehyde-3-phosphate dehydrogenase, aldolase, and phosphofructokinase), deoxyhemoglobin, and some cytoskeletal proteins in its cytosolic domain (Wang, 1994; Hay et al., 2023). The phosphorylation of band 3 can also control the glycolytic process by releasing glycolytic enzymes from the membrane to the cytoplasm (Brunati et al., 2000; Perrotta et al., 2005; Lewis et al., 2009). The interactions between band 3 and deoxyHb also play a critical role in maintaining normal metabolic control (Doctor and Spinella, 2012). However, the impact of Hb oxidation products on the function and integrity of band 3 is not well defined. It has been suggested, however, that Hb oxidation products, hemichromes, have high affinity toward the cytoplasmic domain of band 3 and are reported to mediate oxidative crosslinking through disulfide bonds (Arashiki et al., 2013). Band 3 clustering has been shown to be associated with blebbing and MP generation, which are important characteristics of SCD and β-thalassemias (Mannu et al., 1995; Ferru et al., 2014). Alternative more recent experiments showed that the oxidation product, ferrylHb, and not hemichromes, bind directly with band 3 (Jana et al., 2018; Simons et al., 2018). The complex formation between the ferrylHb and band 3 network of proteins that undergo extensive phosphorylation and ubiquitination leads, in the case of SCD blood (human and mice), to MP formation (Jana et al., 2018; Strader et al., 2020).

In a recent experiment, we assessed total band 3 structural proteins and subsequent phosphorylation in fresh and stored RBCs using specific antibodies against band 3 or against specific phosphotyrosine residues (Y359 and Y21) as well as proteomic analysis. In both cases, we observed no change in band 3 proteins, but an increase in phosphorylation of these sites was noted in the 42-day samples (Hicks et al., under review). RBC’s deformability has been shown to be dependent on band 3 proteins (Lopes de Almeida et al., 2012; Kuo et al., 2021), and it has been shown that RBC’s deformability is increased only when band 3 is dephosphorylated (Wu et al., 2023). This may therefore explain the fact that we detected little or no change in deformability of older RBCs in our current experiments.

Our spectrometric data on oxidative changes in RBCs with age are consistent with those from a recent study in which a variety of biochemical techniques such as vibrational and NanoFTIR spectroscopies were used to elucidate the effect of oxidants on RBCs, including hydrogen peroxide. Changes reported in different degradation processes in membranes and Hb are in keeping with our data obtained with young and old RBCs (Blat et al., 2024).

In this study, we used purified ferryl Hb (by reacting H_2_O_2_ with metHb and rapidly removing the oxidant by chromatography) in a medium of ghost cells (Welbourn et al., 2017). Using specific antibodies against band 3 and Hb, we were able to demonstrate that ferryl Hb generated from freshly prepared RBCs and from 42-day ones. In a complementary experiment where Hb was removed from RBCs (ghost cells) at t = 0 and T = 42 days, little or no changes were seen in protein oxidation as well as band 3 phosphorylation, confirming the need for Hb oxidation intermediates in priming these redox side reactions in both young and more so in 42-day-old RBCs.

A recent large metabolomic study was conducted in which blood from 8,502 healthy blood donors stored for 42 days were investigated under high oxidative hemolysis. The study recommended that the introduction of dietary antioxidant supplements prior to donation can be beneficial (D'Alessandro et al., 2021). We recently reported that including antioxidant such as curcumin with dual effects on Hb oxidative reactions and membrane changes would achieve such benefits in light of our finding in the current investigations (Hicks et al., under review). It follows, therefore, that the development of any countermeasures to control oxidative injuries to RBCs and their biopreservation as a result of biochemical consequences of age-related changes in RBCs should include targeting RBCs’ internal ROS generating sources and Hb oxidative side reactions. As changes are also manifested in membrane alterations, including additives that can target both internal oxidation reactions and membrane changes associated with the aging process may restore oxygen hemostasis of old blood.

In summary, we show in this work that RBC’s oxygenation and oxidation reaction pathways were clearly impacted by the aging process under cold storage conditions. Alterations in iron oxidation impacted RBC’s redox status, oxygen transport, and membrane integrity. Intervention strategies targeting these pathways may ultimately restore oxygen homeostasis in stored blood.

## Data Availability

The raw data supporting the conclusions of this article will be made available by the authors, without undue reservation.
